# Direct autotransfusion in the management of acute pericardial tamponade during catheter ablation for atrial fibrillation: An imperfect but practical method

**DOI:** 10.3389/fcvm.2022.984251

**Published:** 2022-09-23

**Authors:** Xin Zhao, Jian-feng Liu, Xin Su, De-yong Long, Cai-hua Sang, Ri-bo Tang, Rong-Hui Yu, Nian Liu, Chen-xi Jiang, Song-nan Li, Xue-yuan Guo, Wei Wang, Song Zuo, Jian-zeng Dong, Chang-sheng Ma

**Affiliations:** ^1^Department of Cardiology, Beijing Anzhen Hospital, Capital Medical University, Beijing, China; ^2^Department of Cardiology, The Second Medical Center and National Clinical Research Center for Geriatric Diseases, Chinese People's Liberation Army General Hospital, Beijing, China; ^3^Department of Cardiology, The First Affiliated Hospital of Zhengzhou University, Zhengzhou, China

**Keywords:** atrial fibrillation, direct autotransfusion, acute pericardial tamponade, catheter ablation, pericardiocentesis

## Abstract

**Background:**

Acute pericardial tamponade (APT) is one of the most serious complications of catheter ablation for atrial fibrillation (AF-CA). Direct autotransfusion (DAT) is a method of reinjecting pericardial blood directly into patients through vein access without a cell-salvage system. Data regarding DAT for APT are rare and provide limited information. Our present study aims to further investigate the safety and feasibility of DAT in the management of APT during the AF-CA procedure.

**Methods and results:**

We retrospectively reviewed 73 cases of APT in the perioperative period of AF-CA from January 2014 to October 2021 at our institution, among whom 46 were treated with DAT. All included patients successfully received emergency pericardiocentesis through subxiphoid access guided by X-ray. Larger volumes of aspirated pericardial blood (658.4 ± 545.2 vs. 521.2 ± 464.9 ml), higher rates of bridging anticoagulation (67.4 vs. 37.0%), and surgical repair (6 vs. 0) were observed in patients with DAT than without. Moreover, patients with DAT were less likely to complete AF-CA procedures (32/46 vs. 25/27) and had a lower incidence of APT first presented in the ward (delayed presentation) (8/46 vs. 9/27). There was no difference in major adverse events (death/disseminated intravascular coagulation/multiple organ dysfunction syndrome and clinical thrombosis) (0/0/1/0 vs. 1/0/0/0), other potential DAT-related complications (fever/infection and deep venous thrombosis) (8/5/2 vs. 5/3/1), and length of hospital stay (11.4 ± 11.6 vs. 8.3 ± 4.7 d) between two groups.

**Conclusion:**

DAT could be a feasible and safe method to deal with APT during AF-CA procedure.

## Introduction

Acute pericardial tamponade (APT), one of the most serious complications in catheter ablation for atrial fibrillation (AF-CA), usually represents a life-threatening condition requiring emergency pericardiocentesis (1–3). The incidence of APT in AF-CA is approximately 1.0–2.0% (4–6). In a previous global survey, APT-related deaths accounted for a quarter of perioperative deaths of AF-CA (1). In recent years, along with the increasing number of AF-CA worldwide, APT cases have risen rapidly (7). Several reasons might be responsible for APT in the AF-CA procedure: the need for transseptal punctures, the intense and long catheter manipulation in the atrium, using high-power ablation with risk of steam pops, and the need for intense systemic anticoagulation. Although many measures have been taken to prevent APT (for example, the use of transesophageal echocardiography (TEE) or intracardiac ultrasound catheter (ICE) for safer access to the left atrial, Contact Force (CF)–sensing catheter for ablation), APT still has a certain probability. How to deal with APT safely and efficiently is an eternal topic.

In the event of APT, emergency pericardiocentesis is necessary to stabilize hemodynamics and inhibit progressive shock (8). Once pericardial hemorrhage cannot stop, emergency surgical repair may be inevitable. Direct autotransfusion (DAT), a method of reinjecting aspirated pericardial blood directly back into the body via venous access without a cell-salvage system, may be easy to operate and can quickly stabilize hemodynamic status and buy time for anticoagulation reversal or cardiac surgical repair. Previous studies had reported cases of applying DAT to deal with APT during the perioperative period of AF-CA (9–13). However, despite the many potential advantages, DAT has not been conventionally adopted yet, due to the lack of consensus on security. Herein, our present study aims to further investigate the clinical feasibility and safety of DAT in the treatment of APT during AF-CA.

## Methods

### Patients

**Figure 1:**
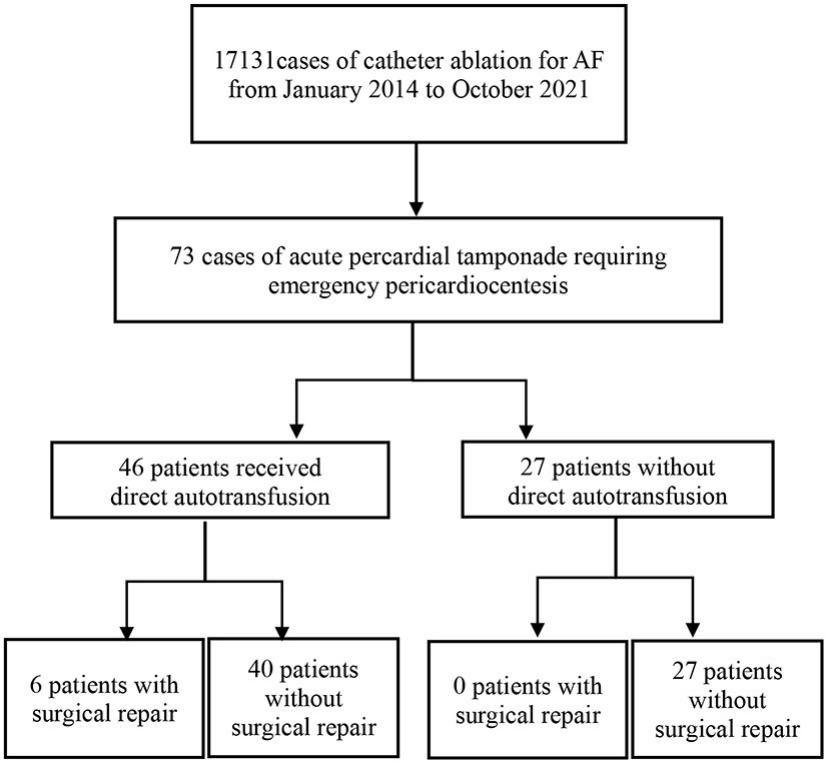
Study flowchart.

Our study retrospectively reviewed APT cases of AF-CA in Beijing Anzhen Hospital Electrophysiological Center from January 2014 to October 2021. During this period, 73 patients suffered from APT, including 46 patients who received DAT without a cell-salvage system (Figure 1).

We collected the demographics, laboratory data, procedure details, and medical histories of all enrolled participants. All subjects signed written consent forms, and the study was approved by the hospital's institutional review board.

### Perioperative anticoagulation

Patients with left atrial and left atrial appendage thrombosis were excluded by TEE 24–48 h before the ablation procedure. Perioperative anticoagulation with warfarin or non-vitamin K antagonist oral anticoagulants (NOACs) was administered as follows: from 2014 to 2018, uninterrupted warfarin strategy or OAC bridging with low-molecular-weight heparin (LMWH) strategy was alternatively performed (about 80% of patients received bridging OAC with LWMH); from 2019 to 2021, an uninterrupted dabigatran strategy was routinely adopted.

During the AF-CA procedure, all subjects were administered unfractionated heparin (100 IU/kg) after transseptal puncture, and intravenous heparin was administered to maintain an activated clotting time (ACT) in excess of 300 s.

### Catheter ablation procedure

All patients underwent AF-CA procedures following a sequential strategy at our institution, which has been well established previously. In brief, one transseptal puncture guided by ICE and/or X-ray was performed routinely in all patients. In patients with paroxysmal AF, pulmonary vein isolation (PVI) was performed with the endpoint of electrical isolation. In patients with persistent AF, along with PVI, additional ablation (such as mitral isthmus line, left atrial roof line, cavotricuspid isthmus line, and superior vena cava) was performed. Carto navigation system was used in nearly 98% of AF ablation procedures, while other navigation systems (Navex and Rhythmia) were only used in very few cases. Moreover, a novel ablation quality marker (Ablation index) was routinely introduced from 2017.

### Management of acute pericardial tamponade and direct autotransfusion

APT was suspected when patients presented with dizziness, vomiting, dyspnea, and/or the systolic blood pressure dropped to <90 mmHg. APT was examined immediately and finally verified by echocardiography, intracardiac echocardiography, and/or fluoroscopy. Once APT was diagnosed, emergency pericardiocentesis through subxiphoid access guided by fluoroscopy was performed by experienced electrophysiologists. Then, a drainage catheter was positioned over the wire into the pericardial cavity to aspirate pericardial blood. Meanwhile, anticoagulation reversal was achieved if necessary, in brief, heparin was reversed by protamine, vitamin K1 was used to reverse warfarin, and dabigatran was reversed by idarucizumab, respectively.

Pericardial blood was aspirated with a separate 60 cc syringe, then transfused immediately via a femoral venous sheath without a cell-salvage system at the operators' discretion. Subsequently, thrombus formation in the syringe was observed, and the pericardial effusion condition was continuously monitored by echocardiography or fluoroscopy. Aspiration and DAT were stopped when pericardial hemorrhage subsided for at least 30 min. If pericardial hemorrhage could not be relieved yet, pericardiotomy and cardiac repair were performed by a cardiac surgery team.

### Perioperative adverse events

Major adverse events included intraprocedural death, clinical thrombosis/adverse thrombotic events (pulmonary embolism, and ischemic stroke), coagulation disorders/disseminated intravascular coagulation (DIC), or multiple organ dysfunction syndrome (MODS). Other complications potentially relevant to DAT were defined as infection/pneumonia, fever, and deep venous thrombosis (DVT).

### Statistical analysis

The data are presented as the means ± standard deviations for continuous variables and frequencies and percentages for categorical variables. To compare the differences between groups, Fisher's exact tests were used for categorical variables, and Mann–Whitney *U* tests were used for continuous variables. Statistical analyses were performed using SPSS 23.0 software (Chicago, IL, USA). All tests were two-tailed, and a *p*-value <0.05 was considered statistically significant.

## Results

### Baseline characteristics

From January 2014 to October 2021, a total of 17131 AF-CA procedures were performed, of which 73 (0.4%) were complicated with APT. The clinical and demographic characteristics of all 73 patients are shown in Table 1. Among them, 46 patients received DAT following emergency pericardiocentesis, whereas the other 27 patients did not receive DAT. There were 40 men (54.8%), and 49 paroxysmal AF (67.1%), and the average age was 65.7 ± 9.8. Moreover, 9 patients presented with congestive heart failure (12.3%), 44 with hypertension (60.3%), 8 with diabetes (11.0%), 8 with previous stroke (11.0%), and 15 with coronary artery disease (20.5%). For periprocedural anticoagulation strategy during AF-CA, 9 received uninterrupted warfarin (12.3%), 23 were applied uninterrupted dabigatran (31.5%), and the other 41 received OAC bridging with LMWH (56.2%). Additionally, 2 patients were on treatment with aspirin (2.7%) and 1 with clopidogrel (1.4%) simultaneously. Compared to patients without DAT, a higher incidence of hypertension (69.6 vs. 44.4%) and OAC bridging with LMWH (67.4 vs. 37.0%) was observed in the DAT group (Table 1).

**Table 1:** Baseline characteristics of the study population.

**Demographic variables**	***n*** **= 73 (total)**	***n*** **= 46 (DAT)**	***n*** **= 27 (non-DAT)**	***p*** **-value**
Age, years	65.7 ± 9.8	65.1 ± 9.6	66.7 ± 10.1	0.348
Male, *n* (%)	40 (54.8)	28 (60.9)	12 (44.4)	0.173
BMI, kg/m^2^	24.2 ± 2.7	24.7 ± 2.8	23.4 ± 2.2	0.079
Type of AF				0.562
Persistent AF, *n* (%)	24 (32.9)	14 (30.4)	10 (37.0)	
Paroxysmal AF, *n* (%)	49 (67.1)	32 (69.6)	17 (63.0)	
Hypertension, *n* (%)	44 (60.3)	32 (69.6)	12 (44.4)	0.034^*^
Diabetes mellitus, *n* (%)	8 (11.0)	5 (10.9)	3 (11.1)	0.975
Coronary artery disease, *n* (%)	15 (20.5)	10 (21.7)	5 (18.5)	0.742
Congestive heart failure, *n* (%)	9 (12.3)	7 (15.2)	2 (7.4)	0.327
Stroke/TIA, *n* (%)	8 (11.0)	6 (13.0)	2 (7.4)	0.457
Baseline laboratory characteristics				
Hemoglobin, g/dl	148.8 ± 13.3	149.1 ± 13.3	148.2 ± 13.7	0.805
Platelet, 10^9^/L	219.3 ± 57.6	221.0 ± 58.5	216.3 ± 57.0	0.710
White blood cell, 10^9^/L	5.6 ± 1.2	5.5 ± 1.1	5.6 ± 1.2	0.770
eGFR, ml/min/1.73 m^2^	89.8 ± 13.9	89.7 ± 13.7	90.0 ± 14.4	0.846
Echocardiographic parameters				
LAD, mm	39.5 ± 5.8	39.8 ± 5.6	39.0 ± 6.3	0.432
LVEDD, mm	46.7 ± 4.4	47.2 ± 4.7	45.8 ± 3.8	0.213
LVEF, %	62.7 ± 6.2	63.2 ± 7.0	61.7 ± 4.5	0.121
CHA_2_DS_2_-VASC score	2.5 ± 1.7	2.5 ± 1.6	2.4 ± 1.9	0.589
HAS-BLED score	1.3 ± 0.9	1.3 ± 0.9	1.2 ± 0.8	0.734
Preoperative anticoagulation therapy				
Warfarin, *n* (%)	9 (12.3)	7 (15.2)	2 (7.4)	0.327
NOACs bridging with LMWH, *n* (%)	41 (56.2)	31 67.4)	10 (37.0)	0.012^*^
Uninterrupted dabigatran, *n* (%)	23 (31.5)	8 (17.4)	15 (55.6)	0.001^*^
Antiplatelet medications				
Aspirin, *n* (%)	2 (2.7)	2 (4.3)	0 (0.0)	0.272
Clopidogrel, *n* (%)	1 (1.4)	1 (2.2)	0 (0.0)	0.440

Values are given as number (percent) or mean ± SD.

AF, atrial fibrillation; BMI, body mass index; eGFR, estimated glomerular filtration rate; LAD, left atrium diameter; LVEDD, left ventricular end-diastolic diameter; LVEF, left ventricular ejection fraction; LMWH, low-molecular-weight heparin; NOAC, non-vitamin K antagonist oral anticoagulant; TIA, transient ischemic attack.

* *p* < 0.05.

### Pericardial tamponade, pericardiocentesis, and direct autotransfusion

During this period, about 50% of patients underwent PVI procedures and the others received more complex procedures. Meanwhile, for all 73 APT patients, 20 patients were performed with PVI only, which is less than those with more complex procedures (53 cases). A sudden onset of APT in the electrophysiological laboratory (EP-lab) was significantly more common in patients with DAT than without DAT (38/46 vs. 9/27). Subsequently, all 73 patients successfully received pericardiocentesis when APT was diagnosed for the first time. There was no significant difference in the application of reversal medications during rescue between two groups (DAT vs. without DAT): vitamin K (3 vs. 1), prothrombin complex (1 vs. 0), and idarucizumab (4 vs. 3), respectively. In addition, more application of protamine sulfate (41 vs. 10), larger volumes of pericardial drainage (611.9 ± 532.7 vs. 262.0 ± 98.4 ml), and higher rates of procedure incompletion (32/46 vs. 25/27) and surgical repair (6 vs. 0) were observed in DAT group (Table 2). It was more frequent in wards of APT patients without DAT, which might be responsible for the less application of protamine.

**Table 2:** Clinical characteristics of 46 cases of direct autotransfusion.

**Case**	**Age/** **Gender**	**AF** **type**	**Anticoagulant**	**Antiplatelet**	**Ablation strategy**	**Blood** **drained** **(ml)**	**Autologous** **blood** **reinfused** **(ml)**	**Reversal** **agent**	**Mechanism** **of** **perforation**	**Surgical** **repair**
1	68/M	PeAF	Warfarin	–	PVI + MAI + CTI + LA roof	300	250	VK1	Unknown	No
2	64/F	PeAF	Warfarin	–	PVI + MAI + CTI + LA roof	310	280	Protamine	Unknown	No
3	75/F	PeAF	LMWH	–	PVI + MAI + CTI + LA roof	240	200	Protamine	Unknown	No
4	52/M	PAF	Warfarin	–	PVI + MAI	350	300	Protamine, VK1	Unknown	No
5	65/M	PAF	Warfarin	–	PVI	230	160	Protamine	Mechanical	No
6	86/M	PAF	LMWH	–	PVI + CTI	200	160	Protamine	Mechanical	No
7	64/F	PAF	LMWH	–	PVI	550	480	Protamine	Unknown	No
8	60/M	PAF	Warfarin	–	PVI	200	180	Protamine	Unknown	No
9	76/F	PAF	LMWH	Aspirin	PVI	450	150	–	Unknown	No
10	71/M	PeAF	LMWH	–	PVI + MAI + CTI + LA roof	190	160	Protamine	Unknown	No
11	60/M	PAF	LMWH	–	PVI + MAI + CTI + LA roof	250	200	Protamine	Unknown	No
12	60/M	PeAF	LMWH	–	PVI + MAI + CTI + LA roof	600	500	Protamine	Unknown	No
13	84/M	PAF	LMWH	–	PVI	1175	1000	Protamine	Unknown	Yes
14	64/F	PAF	LMWH	–	PVI + SVC	210	180	Protamine	Steam pop	No
15	55/M	PeAF	LMWH	Aspirin	PVI + MAI + CTI + LA roof + SVC	180	150	Protamine	Unknown	No
16	74/F	PAF	LMWH	–	PVI	160	130	–	Unknown	No
17	71/M	PAF	LMWH	–	PVI + MAI + LA roof	440	300	Protamine	Steam pop	No
18	77/M	PAF	LMWH	–	PVI	300	250	Protamine	Unknown	No
19	63/F	PeAF	Warfarin	–	PVI + MAI + CTI + LA roof	240	200	Protamine	Unknown	No
20	69/M	PAF	LMWH	–	PVI + MAI + LA roof	1230	1100	Protamine	Mechanical	Yes
21	69/M	PAF	LMWH	–	PVI + CTI + MAI	690	600	Protamine	Unknown	No
22	80/M	PAF	LMWH	–	PVI + MAI	890	830	Protamine	Unknown	No
23	78/F	PAF	LMWH	–	PVI + MAI + LA roof	380	350	Protamine	Unknown	No
24	76/F	PAF	LMWH	–	PVI + MAI + CTI	1860	1550	Protamine	Steam pop	Yes
25	68/M	PAF	LMWH	–	PVI + LA roof	800	720	Protamine	Mechanical	No
26	63/F	PAF	LMWH	–	PVI + LA roof	250	200	Protamine	Unknown	No
27	60/F	PAF	LMWH	–	PVI	926	650	Protamine	Unknown	No
28	61/M	PAF	LMWH	–	PVI + MAI + CTI + LA roof	1300	240	Protamine, PCC	Unknown	No
29	58/M	PeAF	LMWH	–	PVI+MAI + CTI + LA roof	670	600	Protamine	Unknown	No
30	40/M	PAF	LMWH	–	PVI	1030	850	Protamine	Unknown	No
31	75/F	PAF	LMWH	–	PVI + CTI	580	450	Protamine	Steam pop	No
32	60/F	PAF	LMWH	–	PVI + MAI + CTI	330	260	Protamine	Mechanical	No
33	45/M	PAF	LMWH	–	PVI + CTI + SVC	350	300	–	Unknown	No
34	67/F	PAF	Warfarin	–	PVI	500	400	Protamine, VK1	Unknown	No
35	60/F	PAF	LMWH	–	PVI	330	250	Protamine	Steam pop	No
36	62/M	PeAF	LMWH	–	PVI + MAI + CTI + LA roof	1850	1600	Protamine	Steam pop	Yes
37	55/M	PAF	LMWH	–	PVI + MAI + CTI + LA roof	1740	1450	Protamine	Steam pop	Yes
38	71/M	PeAF	Dabigatran	Clopidogrel	PVI + MAI + CTI + LA roof	2350	2100	Protamine,	Unknown	Yes
								Idarucizumab		
39	67/M	PAF	Dabigatran	–	PVI + MAI + CTI + LA roof + SVC	285	240	Protamine	Unknown	No
40	60/M	PAF	Dabigatran	–	PVI	1250	700	Protamine,	Mechanical	No
								Idarucizumab		
41	54/F	PeAF	Dabigatran	–	–	830	700	Protamine,	Mechanical	No
								Idarucizumab		
42	56/M	PAF	Dabigatran	–	PVI	80	60	Protamine	Unknown	No
43	52/M	PeAF	Dabigatran	–	PVI + MAI + CTI + LA roof	300	100	Protamine,	Unknown	No
								Idarucizumab		
44	70/F	PeAF	Dabigatran	–	PVI + CTI	370	300	Protamine	Steam pop	No
45	71/M	PeAF	Dabigatran	–	PVI + MAI + CTI + LA roof	290	250	Protamine	Unknown	No
46	59/F	PAF	LMWH	–	PVI + CTI	110	80	–	Unknown	No

CTI, cavo-tricuspid isthmus; LA, left atrial; LMWH, low-molecular-weight heparin; PAF, paroxysmal atrial fibrillation; PeAF, persistent atrial fibrillation; PCC, prothrombin complex concentrate; PVI, pulmonary vein isolation; SVC, superior vena cava; VK1, vitamin K1.

For all 46 cases with DAT, 7 cases were confirmed to be caused by catheter mechanical operation (15.2%), 8 patients were related to intraoperative steam pop (17.4%), and cumulative ablation effects might be responsible for the other 31 cases. There were 6 (13.0%), 15 (32.6%), and 23 (50.0%) patients with DAT volume >500, >1,000, and <300 ml, respectively (Table 3).

**Table 3:** Comparison of complications between patients with DAT and without-DAT.

**Demographic variables**	**DAT** ***n* = 46**	**Without-DAT** ***n* = 27**	***p*** **-value**
Onset situations, *n* (%)			<0.001^*^
Ward	8 (17.4)	18 (66.7)	
EP-lab	38 (82.6)	9 (33.3)	
Reversal medications, *n* (%)			
Vitamin K	3 (6.5)	1 (3.7)	0.610
Protamine	41 (89.1)	10 (37.0)	<0.001^*^
Idarucizumab	4 (8.7)	3 (11.1)	0.735
Prothrombin complex	1 (2.2)	0 (0)	–
Blood drain volume, ml	611.9 ± 532.7	262.0 ± 98.4	0.001^*^
Autologous blood reinfused, ml	482.4 ± 453.7	–	–
Allogeneic blood transfusion, *n* (%)	9 (19.6)	2 (7.4)	0.161
Complete procedure, *n* (%)	32 (69.6)	25 (92.6)	0.022^*^
Surgical repair, *n* (%)	6 (13.0)	0 (0.0)	0.050
Major adverse events, *n* (%)			
Periprocedural death	0 (0.0)	1 (3.7)	0.370
DIC/MODS/ clinic thrombosis	1 (2.2)	0 (0.0)	0.440
Other complication, *n* (%)			
Fever	8 (17.4)	5 (18.5)	0.903
Infection	5 (10.9)	3 (11.1)	0.975
Deep venous thrombosis	2 (4.3)	1 (3.7)	0.894
Hospital stay, days	11.4 ± 11.6	8.3 ± 4.7	0.598

Direct autotransfusion DAT.

Values are given as number (percent) or mean ± SD.

EP-lab, electrophysiological laboratory; LMWH, low-molecular-weight heparin; PAF, paroxysmal atrial fibrillation; NOAC, non-vitamin K antagonist oral anticoagulant; DIC, disseminated intravascular coagulation; MODS, mutiple organ dysfunction syndrome.

* *p* < 0.05.

### Perioperative adverse event

In the DAT group, 8 patients suffered from infection, and 3 patients developed deep venous thrombosis (DVT) of the lower limbs following the procedure. However, There was no significant difference in major adverse events (death, DIC/MODS/clinical thrombosis) (0/0/1/0 vs. 1/0/0/0), other complications potentially relevant to DAT (fever, infection, and deep venous thrombosis) (8/5/2 vs. 5/3/1), and length of hospital stay (11.4 ± 11.6 vs. 8.3 ± 4.7 d) between two groups (DAT vs. without DAT). Unfortunately, even if pericardiocentesis was successfully performed, 1 patient died of brain death in a group without DAT (Table 3).

## Discussion

To the best of our knowledge, the present study represented the largest single-center series referring to the feasibility and safety of DAT in the management of APT during the AF-CA procedure. We observed larger volumes of drained blood reinfused and more need for surgical repair in patients with DAT. Although several complications were recorded, there was no significance in major adverse events and other potential DAT-related complications between patients with and without DAT. Our study demonstrated that DAT might be not perfect, but a feasible, effective/efficient, and safe method in the management of APT during the AF-CA procedure.

APT is one of the most serious complications during AF-CA and may occur at any time for a variety of reasons, including steam pop, excessive radiofrequency energy, mechanical perforation, or transseptal puncture. Moreover, systemic anticoagulation during perioperative AF-CA is also an important risk factor for pericardial tamponade. In the past few years, with AF-CA procedures gradually extending to low-volume centers, the overall incidence of APT had shown an increasing trend: from 0.74% in 2000 to 2.24% in 2010 (14).

In recent years, new catheter, technology, and concept for AF-CA and perioperative anticoagulation strategies had been innovated, including CF-sensing catheter, intracardiac ultrasound catheter, high-power ablation, ablation index (15), Near-Zero X-ray technology (16) and uninterrupted oral anticoagulation. Although the Smart AF Trial (17) showed a high incidence of APT in AF-CA procedure (2.5%, 4/161 patients) with CF-sensing catheter. Toccastar trial (18) demonstrated that the APT incidence when using CF-sensing catheters was lower; however, there was no difference compared to non-sensing catheters (0.66 vs. 0.70%). Performing left atrial mapping, ICE, and CF-sensing catheter is expected to reduce the rate of APT; however, applying aggressive ablation strategy, increasing ablation intensity, and gradual expansion of AF-CA to low-volume centers may increase the possibility of APT. So herein, the advance of technologies, catheter, and strategies has not reduced the incidence of APT while coping with more complex procedures and improving the success rate of AF-CA.

It is crucial to identify pericardial hemorrhage as soon as possible, which facilitates the prevention of the progression from effusion to tamponade and reverses this fatal complication. The cessation of spontaneous pericardial hemorrhage is unpredictable and depends on a variety of factors, including the size and site of the perforation, pericardial pressure, perforation properties, and perioperative anticoagulation condition (19). Emergency surgical repair is inevitable if bleeding continues after pericardiocentesis, reversal of anticoagulation, and blood transfusion. When APT developed, following emergency pericardiocentesis, rescuing measures including blood transfusion are usually performed in the first place to stabilize hemodynamics and buy time for surgical repair.

Blood-cell salvage system can separate red blood cells from cell debris, fat particles, activated cytokines, clotting factors, and platelets, which may reduce autotransfusion-related complications (20, 21). Current data concerning salvage autotransfusion mainly came from surgical operations, such as cardiac surgery and traumatic surgery (22). Due to the potential risks of thromboembolism and infection, several blood filtering methods reducing the potential complications had been established (23–25). However, the extensive use of cell salvage (especially for retransfused blood >1,500 ml) might lead to a critical loss of coagulation factors and platelets, resulting in a bleeding diathesis (9, 26, 27). To sum up, the results of clinical trials had shown controversial clinical benefits of the cell-salvage system during the intraoperative period (20, 22, 25). For AF-CA, Venkatachalam et al. (28) reported 9 cases of APT successfully treated with autologous blood transfusion via cell-salvage system. Nevertheless, a cell-salvage system usually required more time to prepare and was not available in all institutions, especially in low-volume centers, which might make a difference in this fatal complication.

DAT is a timely and accessible method of reinjecting pericardial blood directly back to the body through vein access. Pericardiocentesis combined with DAT can quickly stabilize hemodynamic status, reduce allogeneic blood transfusion, and more importantly, buy time for patients who ultimately require surgical repair (9, 27). Theoretically, DAT will be effective in the treatment of APT for it can be initiated immediately and with minimal preparation. However, DAT has not been routinely adopted by most interventional electrophysiologists due to its potential risks of thrombotic and infective events. Recently, two studies (10, 11) preliminary investigated the feasibility of DAT in APT during the perioperative period of EP procedures and revealed that DAT reduced the need for allotransfusion and surgical repair without increasing major complications. However, among different EP procedures, only a few (<10) AF patients were enrolled in either study, therefore, their conclusions cannot be completely applicable for AF-CA and need to be further explored. Our present study involving 46 patients with DAT in AF-CA procedures showed a lower incidence of surgical repair (13%) without major adverse events. Despite several cases of infection and DVT being recorded, there were no statistical differences between patients with and without DAT. In our opinion, such complications might be attributed to multiple factors, for instance: pericardial tamponade itself, pericardiocentesis, stress response, extended postoperative bed rest, and surgical operations, instead of the DAT part.

However, several differences in terms of autotransfusion in different scenarios (surgery or EP procedures) should be noted. Firstly, when traditional surgical procedures are performed, the cell-salvage system is usually prepared in advance or in the preparation operation room. However, APT during the EP procedures is usually emergent and unpredictable to prepare a cell-salvage instrument. Secondly, surgical procedures are usually accompanied by massive tissue damage, and the restored blood flow may be rich in tissue/cell debris, small embolism, and/or contaminants. However, catheter-based cardiac procedures usually lead to local hemorrhage, which generates little tissue/cell debris. Moreover, as a closed cavity, the pericardium is basically not involved in bacterial infections. Lastly, for atria are low-pressure cavities, pericardial hemorrhage in AF-CA is usually slower and less in amount than other types of cardiac interventional procedures (such as transcatheter aortic valve implantation and percutaneous coronary intervention), which means that it is not easy to form thrombi in aspirated blood due to the fibrinolytic property of the pericardium. Therefore, DAT may not increase clinical complications including embolism, especially when the transfusion volume is relatively small.

## Limitations

Several limitations should be considered. First, although more cases were enrolled, our present study was still a retrospective single-center study. The safety of DAT needs to be further validated by randomized trials and pathophysiological studies on blood. Besides, only 50% of patients had a reinjected blood volume >300 ml, which might lead to a bias. However, as an emergent and fatal complication, APT is usually unpredictable and needs to be treated with close observation.

## Conclusion

Our present study showed that direct autotransfusion of pericardial drainage blood in the management of APT during AF-CA procedure could be a safe, efficient, and feasible method. Although DAT might not be a perfect method for APT, when the primary risk is immediate death, any life-saving measures should be worth considering. In the clinical practice of dealing with APT, DAT could be safely applied, especially for patients with the expected large amount of bleeding and unstable hemodynamics and hemorrhage difficult to judge, which limits the need for transfusions and buy the time for cardiac surgical repair.

## Data availability statement

The original contributions presented in the study are included in the article/supplementary material, further inquiries can be directed to the corresponding author/s.

## Ethics statement

The studies involving human participants were reviewed and approved by Beijing Anzhen Hospital, Capital Medical University. The patients/participants provided their written informed consent to participate in this study.

## Author contributions

D-yL, R-bT, R-HY, and C-hS defined the theme of review. XZ and J-fL wrote the manuscript. XS, NL, C-xJ, X-yG, WW, S-nL, and SZ took part in preparing the manuscript. XZ, J-fL, J-zD, and C-sM prepared and reviewed the manuscript before publication. All authors confirmed that they have read and approved the manuscript and they have met the criteria for authorship.

## Funding

This work was supported by grants from the National Natural Science Foundation of China (81600262, 81530016, 81670291, and 81870244).

## Conflict of interest

The authors declare that the research was conducted in the absence of any commercial or financial relationships that could be construed as a potential conflict of interest.

## Publisher's note

All claims expressed in this article are solely those of the authors and do not necessarily represent those of their affiliated organizations, or those of the publisher, the editors and the reviewers. Any product that may be evaluated in this article, or claim that may be made by its manufacturer, is not guaranteed or endorsed by the publisher.

## References

[1] Cappato R Calkins H Chen SA Davies W Iesaka Y Kalman J. Prevalence and causes of fatal outcome in catheter ablation of atrial fibrillation. J Am Coll Cardiol. (2009) 53:1798–803. 10.1016/j.jacc.2009.02.022 19422987

[2] Doppalapudi H Yamada T Kay GN. Complications during catheter ablation of atrial fibrillation: identification and prevention. Heart Rhythm. (2009) 6:S18–25. 10.1016/j.hrthm.2009.07.025 19864189

[3] Calkins H Kuck KH Cappato R Brugada J Camm AJ Chen SA. HRS/EHRA/ECAS expert consensus statement on catheter and surgical ablation of atrial fibrillation: recommendations for patient selection, procedural techniques, patient management and follow-up, definitions, endpoints, and research trial design: a report of the Heart Rhythm Society (HRS) Task Force on Catheter and Surgical Ablation of Atrial Fibrillation. Developed in partnership with the European Heart Rhythm Association (EHRA), a registered branch of the European Society of Cardiology (ESC) and the European Cardiac Arrhythmia Society (ECAS); and in collaboration with the American College of Cardiology (ACC), American Heart Association (AHA), the Asia Pacific Heart Rhythm Society (APHRS), and the Society of Thoracic Surgeons (STS) Endorsed by the governing bodies of the American College of Cardiology Foundation, the American Heart Association, the European Cardiac Arrhythmia Society, the European Heart Rhythm Association, the Society of Thoracic Surgeons, the Asia Pacific Heart Rhythm Society, and the Heart Rhythm Society. Heart Rhythm. (2012) 9:632–96. 10.1016/j.hrthm.2011.12.016 22386883

[4] Cappato R Calkins H Chen SA Davies W Iesaka Y Kalman J. Updated worldwide survey on the methods, efficacy, and safety of catheter ablation for human atrial fibrillation. Circ Arrhythm Electrophysiol. (2010) 3:32–8. 10.1161/CIRCEP.109.859116 19995881

[5] Hamaya R Miyazaki S Taniguchi H Kusa S Nakamura H Hachiya H. Management of cardiac tamponade in catheter ablation of atrial fibrillation: single-centre 15 year experience on 5222 procedures. Europace. (2018) 20:1776–82. 10.1093/europace/eux307 29161368

[6] Cappato R Calkins H Chen SA Davies W Iesaka Y Kalman J. Worldwide survey on the methods, efficacy, and safety of catheter ablation for human atrial fibrillation. Circulation. (2005) 111:1100–5. 10.1161/01.CIR.0000157153.30978.67 15723973

[7] Raymond-Paquin A Andrade J Macle L. Catheter ablation: an ongoing revolution. J Thorac Dis. (2019) 11:S212–5. 10.21037/jtd.2019.02.20 30997179 PMC6424795

[8] Tsang TS Freeman WK Barnes ME Reeder GS Packer DL Seward JB. Rescue echocardiographically guided pericardiocentesis for cardiac perforation complicating catheter-based procedures. The Mayo Clinic experience. J Am Coll Cardiol. (1998) 32:1345–50. 10.1016/S0735-1097(98)00390-8 9809946

[9] Larsen TR Huizar JF. Direct autologous blood transfusion in cardiac tamponade: where safety is not always first. J Cardiovasc Electrophysiol. (2019) 30:1294–6. 10.1111/jce.14048 31240789

[10] Barbhaiya CR Guandalini GS Jankelson L Park D Bernstein S Holmes D. Direct autotransfusion following emergency pericardiocentesis in patients undergoing cardiac electrophysiology procedures. J Cardiovasc Electrophysiol. (2020) 31:1379–84. 10.1111/jce.14462 32243641

[11] Beyls C Hermida A Duchateau J Maury P Taieb J Laurent G. Management of acute cardiac tamponade by direct autologous blood transfusion in interventional electrophysiology. J Cardiovasc Electrophysiol. (2019) 30:1287–93. 10.1111/jce.14050 31240813

[12] Fiocca L Cereda AF Bernelli C Canova PA Serino F Niglio T. Autologous blood reinfusion during iatrogenic acute hemorrhagic cardiac tamponade: Safety and feasibility in a cohort of 30 patients. Catheter Cardiovasc Interv. (2019) 93:E56–62. 10.1002/ccd.27784 30244510

[13] Huang XM Hu JQ Zhou F Qin YW Cao J Zhou BY. Early diagnosis and rescue pericardiocentesis for acute cardiac tamponade during radiofrequency ablation for arrhythmias. Is fluoroscopy enough? Pacing. Clin Electrophysiol. (2011) 34:9–14. 10.1111/j.1540-8159.2010.02938.x 21029133

[14] Deshmukh A Patel NJ Pant S Shah N Chothani A Mehta K. In-hospital complications associated with catheter ablation of atrial fibrillation in the United States between 2000 and 2010: analysis of 93 801 procedures. Circulation. (2013) 128:2104–12. 10.1161/CIRCULATIONAHA.113.003862 24061087

[15] Reinsch N Futing A Buchholz J Ruprecht U Neven K. Influence of ablation index on the incidence of cardiac tamponade complicating pulmonary vein isolation. Herz. (2021) 46:228–34. 10.1007/s00059-020-04988-y 33026484

[16] Mascia G Giaccardi M A. New era in zero x-ray ablation. Arrhythm Electrophysiol Rev. (2020) 9:121–7. 10.15420/aer.2020.02 33240507 PMC7675142

[17] Natale A Reddy VY Monir G Wilber DJ Lindsay BD McElderry HT. Paroxysmal AF catheter ablation with a contact force sensing catheter: results of the prospective, multicenter SMART-AF trial. J Am Coll Cardiol. (2014) 64:647–56. 10.1016/j.jacc.2014.04.072 25125294

[18] Reddy VY Dukkipati SR Neuzil P Natale A Albenque JP Kautzner J. Randomized, controlled trial of the safety and effectiveness of a contact force-sensing irrigated catheter for ablation of paroxysmal atrial fibrillation: results of the tacticath contact force ablation catheter study for atrial fibrillation (TOCCASTAR) study. Circulation. (2015) 132:907–15. 10.1161/CIRCULATIONAHA.114.014092 26260733

[19] Mujovic N Marinkovic M Markovic N Kocijancic A Kovacevic V Simic D. Management and outcome of periprocedural cardiac perforation and tamponade with radiofrequency catheter ablation of cardiac arrhythmias: a single medium-volume center experience. Adv Ther. (2016) 33:1782–96. 10.1007/s12325-016-0402-x 27554091 PMC5055551

[20] Baker RA Merry AF. Cell salvage is beneficial for all cardiac surgical patients: arguments for and against. J Extra Corpor Technol. (2012) 44:P38-41. 22730871 PMC4557446

[21] Maleck WH Petroianu GA. Autologous Blood Transfusion. (1999) p. 154. 10.1093/bja/82.1.154 10325860

[22] Wang G Bainbridge D Martin J Cheng D. The efficacy of an intraoperative cell saver during cardiac surgery: a meta-analysis of randomized trials. Anesth Analg. (2009) 109:320–30. 10.1213/ane.0b013e3181aa084c 19608798

[23] Murphy GJ Allen SM Unsworth-White J Lewis CT Dalrymple-Hay MJ. Safety and efficacy of perioperative cell salvage and autotransfusion after coronary artery bypass grafting: a randomized trial. Ann Thorac Surg. (2004) 77:1553–9. 10.1016/j.athoracsur.2003.10.045 15111142

[24] Djaiani G Fedorko L Borger MA Green R Carroll J Marcon M. Continuous-flow cell saver reduces cognitive decline in elderly patients after coronary bypass surgery. Circulation. (2007) 116:1888–95. 10.1161/CIRCULATIONAHA.107.698001 17923575

[25] Rubens FD Boodhwani M Mesana T Wozny D Wells G Nathan HJ. The cardiotomy trial: a randomized, double-blind study to assess the effect of processing of shed blood during cardiopulmonary bypass on transfusion and neurocognitive function. Circulation. (2007) 116:I89–97. 10.1161/CIRCULATIONAHA.106.678987 17846332

[26] Gao LY Tang RB Dong JZ Liu XP Long DY Yu RH. Autotransfusion in the management of cardiac tamponade occurring during catheter ablation of atrial fibrillation. Chin Med J. (2010) 123:961–3. 20497695

[27] O'Neill MD Jais P Derval N Hocini M Haissaguerre M. Two techniques to avoid surgery for cardiac tamponade occurring during catheter ablation of atrial fibrillation. J Cardiovasc Electrophysiol. (2008) 19:323–5. 10.1111/j.1540-8167.2007.00973.x 17900253

[28] Venkatachalam KL Fanning LJ Willis EA Beinborn DS Bradley DJ Cha YM. Use of an autologous blood recovery system during emergency pericardiocentesis in the electrophysiology laboratory. J Cardiovasc Electrophysiol. (2009) 20:280–3. 10.1111/j.1540-8167.2008.01313.x 19261039

